# Reduction of cytosolic phospholipase A_2_α upregulation delays the onset of symptoms in SOD1G93A mouse model of amyotrophic lateral sclerosis

**DOI:** 10.1186/s12974-016-0602-y

**Published:** 2016-06-01

**Authors:** Yulia Solomonov, Nurit Hadad, Rachel Levy

**Affiliations:** Immunology and Infectious Diseases Laboratory, Department of Clinical Biochemistry and Pharmacology, Faculty of Health Sciences, Ben-Gurion University of the Negev and Soroka University Medical Center, Beer-Sheva, Israel

**Keywords:** Cytosolic phospholipase A_2_α, Motor neurons, Motor function, Glia, Astrocytes

## Abstract

**Background:**

Amyotrophic lateral sclerosis (ALS) is a fatal multifactorial neurodegenerative disease characterized by selective death of motor neurons in the cortex, brainstem, and spinal cord. Cytosolic phospholipase A_2_ alpha (cPLA_2_α) upregulation and activation in the spinal cord of patients with sporadic ALS and in the spinal cord of human mutant SOD1G93A (hmSOD1) transgenic mice were recently reported.

**Methods:**

cPLA_2_α upregulation in the brainstem and spinal cord was reduced by brain infusion of a specific antisense oligonucleotide against cPLA_2_α (AS), and the effect was evaluated on disease progression and brain cell activation.

**Results:**

We found that the elevation of cPLA_2_α protein expression in the spinal cord was first detected at 6-week-old hmSOD1 mice and remained elevated during their whole life span. Reduction of the elevated expression of cPLA_2_α in the spinal cord of hmSOD1 mice by brain infusion of an AS at week 15 (shortly before the appearance of the disease symptoms), for a duration of 6 weeks, delayed the loss of motor neuron function in comparison with hmSOD1 mice and with sense brain-infused hmSOD1 mice. To characterize the effect of cPLA_2_α upregulation on different processes taking place at the appearance of the disease symptoms, mice were brain infused with AS or with sense at week 15 for 3–4 weeks. The AS treatment that reduced cPLA_2_α upregulation in the spinal cord of AS-treated hmSOD1 mice (as analyzed at week 18–19) prevented the reduction in the number of the neurons (detected by NeuN) and inhibited astrocyte activation (detected by GFAP) and microglia activation (detected by Iba-1 and by CD40). In addition, AS treatment blunted the upregulation of the proinflammatory enzyme-inducible nitric oxide synthase (iNOS) and cyclooxygenase-2 (COX-2) detected in hmSOD1 mice.

**Conclusions:**

Since specific reduction of cPLA_2_α in the brainstem and spinal cord significantly attenuated the development of the disease, cPLA_2_α may offer an efficient target for treatment of ALS.

## Background

Amyotrophic lateral sclerosis (ALS) is a severe degenerative disorder, mainly affecting the motor neurons in the spinal cord, brainstem, and cortex. Most of the cases (about 90 %) are sporadic. Familial cases have been linked to mutations in a number of genes, including Cu/Zn superoxide dismutase (SOD1), TAR DNA binding protein (TDP-43), and chromosome 9 open reading frame 72 (C9ORF72) repeat expansions [1]. Mutant SOD1 is the best characterized form of familial ALS, accounting for 15–20 % of familial cases [2]. The etiology of sporadic ALS is unknown, although it is generally believed that sporadic and familial ALS may share pathological mechanisms. The pathophysiology of the multifactorial-multisystemic ALS disease includes various mechanisms. Among them are oxidative damage, high levels of glutamate, a reduced secretion of neurotrophic factors, protein aggregations, malfunctioning of the mitochondria, rupture in the axonal passage, destruction in the calcium metabolism, and changes in the skeletal proteins [3]. Although ALS is not primarily considered an inflammatory or immune-mediated disease, immune mechanisms appear to play a role in the pathogenesis of the disease. In both ALS patients and animal models, inflammatory responses have been observed [3–5]. Microglia [6] and astrocytes [7] are activated during the progression of the disease, and evidence suggests that they contribute to neuronal death. Selective ablation of mutant SOD1 in astrocytes and microglial cells by conditional deletion [8] and neonatal wild-type bone marrow transplantation [6] increased motor neuron survival and life span. It has also been reported that in the CNS, natural killer cells and peripheral T cells infiltrate the spinal cord [9].

Previous findings suggested that cytosolic phospholipase A_2_α (cPLA_2_α) is regarded as an important source of inflammation. cPLA_2_α specifically hydrolyzes phospholipids containing arachidonic acid at the sn-2 position [10, 11] and is generally thought to be the rate-limiting step in the generation of eicosanoids and platelet-activating factor. These lipid mediators play critical roles in the initiation and modulation of inflammation and oxidative stress. cPLA_2_α is ubiquitous in brain cells and is essential for their physiological regulation. However, elevated cPLA_2_α expression and activity were detected in the inflammatory sites in a vast array of inflammatory diseases, including neurodegenerative diseases [12–14]. Increased expression and activity of cPLA_2_α have been detected in neurons, in astrocytes, and in microglia in the spinal cord, brainstem, and cortex of sporadic ALS patients [15] and in the spinal cord of G93A human mutant transgenic (hmSOD1) mice [16], suggesting that cPLA_2_α may have an important role in the pathogenesis of the disease in all ALS patients. Due to the complexity of biological pathways involved in ALS, it is now thought that blunting several aspects of the disease may offer distinct advantages over targeting one pathway. Since our previous studies showed that elevated cPLA_2_α is involved in glia activation, oxidative stress [17] and neuronal apoptotic death [18], targeting cPLA_2_α may affect these processes that have been shown to be involved in the pathogenesis of ALS. The present study aims to determine whether the elevation of cPLA_2_α protein expression in the spinal cord and brainstem has a role in the pathogenesis of the disease, using a mouse model of hmSOD1 mice.

## Methods

### Animals

B6.Cg-Tg(SOD1G93A)1Gur/J hemizygous transgenic male mice were obtained from Jackson Laboratory (Bar Harbor, ME, USA). The hemizygous transgenic male mice were also obtained by mating hemizygous transgenic males with C57BL/6J females (Jackson Laboratory) so that each litter would generate hemizygous SOD1G93A transgenic mice and littermate wild-type controls. Transgenic male offsprings were genotyped by PCR assay of DNA obtained from tail tissue (according to Jackson Laboratory). The study included male mice to avoid estrogen effect. The infusion of anti-cPLA_2_α antisense oligonucleotide or corresponding sense to mice (were performed as described in our previous study [19] and according others [20–22]). A microosmotic pump (Alzet, Durect Corp. Cupertino, CA, USA) that had been filled with oligonucleotide and incubated overnight in saline at 37 °C was implanted in a subcutaneous pocket. The pump that was attached to a cannula (Alzet brain infusion kit 3, Durect Corp. Cupertino, CA, USA) stereotaxically implanted into the right lateral cerebral ventricle (−1.0 mm mediolateral and −0.5 mm anteroposterior from Bregma). The study was approved by Ben-Gurion University Institutional Animal Care and Use Committee (IL-37-05-2012) and was conducted according to the Israeli Animal Welfare Act following the guidelines of the Guide for Care and Use of Laboratory Animal (National Research Council, 1996).

### Antisense oligonucleotide against cPLA_2_α

Antisense oligonucleotides against cPLA_2_α were engineered using the computer-based approach RNADraw V1.1 (Mazura Multimedia, Stockholm, Sweden). An oligo-deoxynucleotide antisense (tcaaaggtctcattccaca) and its corresponding sense with phosphorothioate modifications on the last 3 bases at both 5′ and 3′ ends were used as described in our previous article [19]. The specificity to cPLA_2_α was analyzed by blast search program and was demonstrated in our previous study [23]. Mice (around 25 g of weight) received 10 μg/day phospho oligo-deoxynucleotides diluted in saline. The antisense concentration used in the study reduced cPLA_2_α levels in the brainstem and spinal cord of ALS mice to the level detected in wild-type mice.

### Brain stem and spinal cord tissue preparation

Mice were deeply anesthetized and transcardially perfused with 20 ml of PBS [24].

#### For immunoblot analysis

Spinal cords were harvested in lysis buffer containing 50 mM Tris pH = 8, 150 mM NaCl, 1 % NP-40, 0.5 % DOC, 0.1 % SDS, 10 μg/ml leupeptin, 1 mM phenylmethylsulfonyl fluoride, 10 μg/ml aprotinin, 1 mM benzamidine, 20 mM P-nitrophenyl phosphate, 5 mM Na_3_VO_4_,10 mM NaF, and 50 mM β-glycerol phosphate. Soluble extracts were prepared by centrifugation at 13,000 g for 20 min at 4 °C. Lysate protein was separated on SDS-PAGE electrophoresis and transferred to nitrocellulose membranes. Membranes were incubated in Tris-buffered saline (10 mM Tris, 135 mM NaCl, pH 7.4), with 0.1 % Tween 20 (TBS-T) containing 5 % non-fat milk for 1.5 h at 25 °C. The blots were then incubated with primary antibodies: 1:1000 rabbit polyclonal anti-cPLA_2_α against the carboxyterminal residues (Cell Signaling Technology, Beverly, MA, USA), 1:1000 rabbit anti-COX-2 (Abcam, Cambridge, UK), 1:1000 rabbit anti-iNOS (Cayman chemical, Ann Arbor, MI, USA), 1:500 rabbit anti-calreticulin (Thermo Scientific, Rockford, IL, USA) as primary antibodies for overnight at 4 °C. After washing with TBS-T, they were incubated with secondary antibody peroxidase-conjugated goat anti-rabbit (Amersham Biosciences, Buckinghamshire, UK) for 1 h at 25 °C and developed using the enhanced chemiluminescence (ECL) detection system (PerkinElmer, Waltham, MA, USA). Proteins were quantified using video densitometry analysis (ImageJ version 4.0 Fuji).

#### For immunostaining

The brainstem or spinal cord were fixed [25] in paraformaldehyde 4 %/PBS solution overnight at 4 °C. The brainstem or spinal cord were then transferred to PBS containing 30 % sucrose for 24 h and then embedded in a 1:2 mixture of 30 % sucrose in PBS/Tissue-Tek OCT (VWR, Radnor, PA, USA), frozen in liquid nitrogen and stored at −80 °C. Sections were made by cryostat (Leica Biosystems, Vienna, Austria) at 12-μm thickness, washed in PBS/Tween 0.05 %, incubated in PBS/glycine 0.1 % for 5 min, and incubated in a blocking solution (3 % normal donkey serum and 2 % BSA) at room temperature for 1 h. Then, these sections were incubated with primary antibodies diluted in blocking solution overnight at 4 °C. The primary antibodies used in the study were 1:100 rabbit anti-cPLA_2_ (Santa Cruz Biotechnology, Santa Cruz, CA, USA), 1:100 rabbit anti-pcPLA_2_α (Cell Signaling Danvers, MA USA), 1:1000 rabbit anti-Iba-1 (Wako Chemicals Richmond, VA, USA), 1:500 rabbit anti-GFAP (Dako Glostrup Denmark), 1:100 mouse anti-NeuN (Millipore Darmstadt, Germany), and 1:100 rat anti-CD40 or 1:100 rat anti-CD169 (AbD Serotec, Kidlington, UK). Sections were washed with PBS/Tween 0.05 % and incubated with 1:200 Cy3 or 1:100 anti-mouse Alexa488 or anti-rabbit DyLight-conjugated secondary antibodies (Jackson Immunoresearch Laboratories, West Grove, PA, USA) for 1 h at room temperature. For double staining, DyLight was used as a secondary antibody for cPLA_2_α (green) and 1:25 goat anti-Iba-1 (Novus Biologicals Littleton, USA) or 1:100 mouse anti-GFAP (Millipore Darmstadt, Germany) with Cy3 were used as second antibodies (red). The staining of samples from the different treatments was performed in parallel. For each treatment, a negative control was prepared by omitting the primary antibody. Sections were mounted with anti-fading mounting medium (Electron Microscopy Sciences (EMS), Hatfield, PA, USA) and photographed in a blinded fashion using a fluorescent microscope (Olympus, BX60, Hamburg, Germany) or with confocal microscopy (Olympus, FluoView 1000, Tokyo, Japan). Using a confocal microscope, Z-sections were taken at 0.5-μm intervals and the results present Z-stack images. Fluorescence intensity was determined for cPLA_2_α using CellProfiler program. The percentage of fluorescence intensity of the cell area was determined for the different cell types using CellProfiler program.

### Motor function measurement by rotarod

A rotarod test was used to evaluate the motor performance of the mice using an accelerating paradigm of 0.12 rpm/s. After a learning period of several days, mice were able to stay on the rotarod (Rotamex-5, Columbus instruments, Columbus, OH, USA) for up to 150 s. Each mouse was given 3 trials and the best performance was used as a measure for motor neuron ability. Mice were tested twice a week from age of 7 weeks until they could no longer perform the task.

### Neurological score evaluation by ladder testing

To evaluate the animal’s overall neurological state, we performed a neurological scoring based on the ladder test described in details by others [26], which is an extension of the hanger test. Shortly, the ladder was placed at a 45° angle; after a brief training period, healthy mice quickly and efficiently climb up the ladder. As the disease progresses, the animals’ ability to climb was hindered beginning with leg tremors and developing up until the point where the mice can simply not climb up the ladder at all. Mice were evaluated for their neurological score three times a week by a blinded observer. The mice were scored based on their performance on the ladder test with a score of 12 representing completely healthy mice and 0 correlating with disease end stage, described in details [26].

### End-stage analysis

To determine the mortality in a reliable and human fashion, end stage was defined as the inability of the mice to right themselves 30 s after being placed on one of their sides.

### Statistical analysis

Data were expressed as mean ± standard error of the mean (SEM). Significance was determined by either one- or two-way analysis of variance (ANOVA) followed by a posteriori Bonferroni’s test for multiple comparisons provided by GraphPad Prism version 5.00 for Windows (GraphPad Software, San Diego, CA, USA). Survival was evaluated by Kaplan-Meier analysis.

## Results

**Fig. 1:**
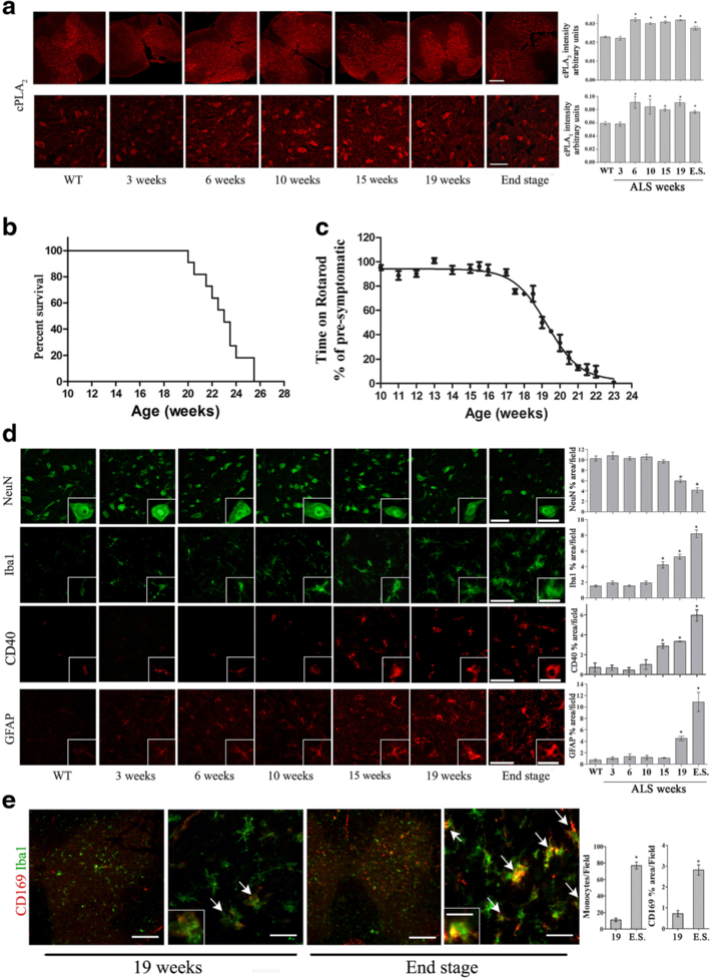
cPLA_2_α protein levels in the spinal cord of hmSOD1 mice during the development of the disease in hmSOD1 mice. **a** Representative cPLA_2_α protein expression in the spinal cord tissue sections along the hmSOD1 mouse life span. Scale bars = 250 μm for upper section and 50 μm for lower sections. The mean ± SEM of the cPLA_2_α fluorescence intensity is presented in the bar graph (*n* = 8 mice for each time point, five fields were analyzed for each mouse). *p < 0.001—significance from WT mice. **b**, **c** Development of the disease monitored by Kaplan-Meier survival curve and motor performance on accelerating rotarod test (*n* = 12 mice in each group). **d** Representative spinal cord sections of neurons (NeuN), microglia (Iba-1 and CD40), and astrocytes (GFAP) during the hmSOD1 mouse life span. The mean ± SEM of percentage of the cell area is presented in the bar graphs (*n* = 8 for each time point, five fields were analyzed for each mouse). Scale bars = 50 μm and in the inset 20 μm. *p < 0.001—significance from WT mice. **e** Representative staining of peripheral monocytes detected by double staining of Iba-1 and CD169, shown by *arrows*. Scale bars = 250 μm for *left section* and 50 μm for *right section* in each pair. Scale bars in the insets = 20 μm. The mean ± SEM of number of monocytes and of percentage of the cell area is presented in the bar graphs (*n* = 8 for each time point, five fields were analyzed for each mouse). *p < 0.001—significance in comparison to week 19

**Fig. 2:**
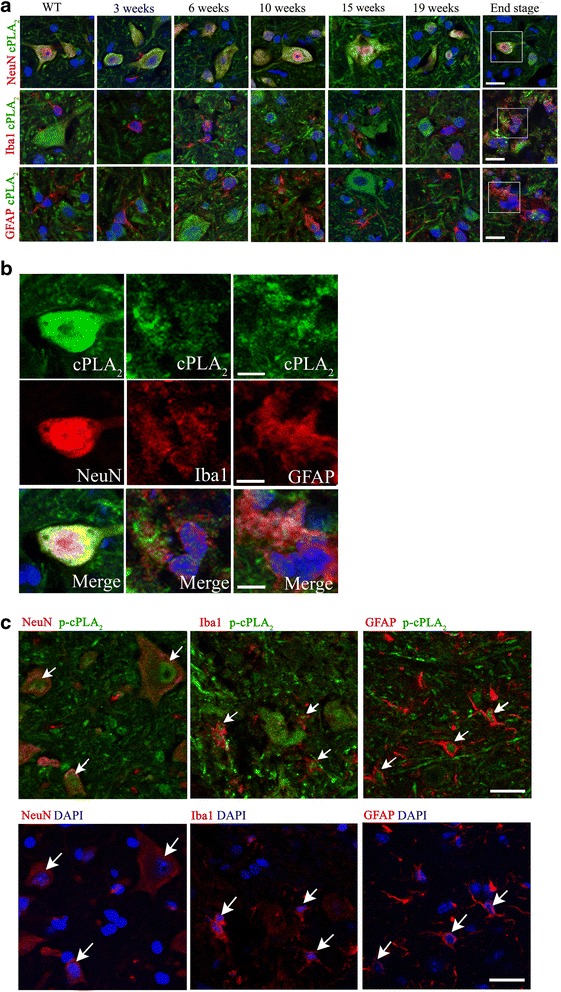
Elevated cPLA_2_α protein expression in the different cell types of the spinal cord. **a** Representative pictures of immunofluorescence double staining of cPLA_2_α (*green*) with motor neurons (NeuN), microglia (Iba-1), or astrocytes (GFAP) in the spinal cord during hmSOD1 mouse life span (out of 8 mice in each time point, presented in Fig. 1a). DAPI staining for cell nuclei. Scale bars = 20 μm. **b** Insets of the immunofluorescence double staining of cPLA_2_α with motor neurons, microglia, or astrocytes (presented in **a**), in a higher magnification. Scale bars = 20 μm. **c** Immunofluorescence double staining of phospho-cPLA_2_α (p-cPLA_2_) and motor neurons (NeuN), microglia (Iba-1) and astrocytes (GFAP). Shown representative immunofluorescence confocal microscopy pictures (out of 8 mice in each group). *Lower panel* presents the cell staining and DAPI to show the cell nuclei. Scale bars = 20 μm

In order to study the role of cPLA_2_α in the development of ALS, cPLA_2_α protein expression was analyzed in the spinal cord of hmSOD1 mice (Fig. 1a) at different stages during their life span (Fig. 1b). Immunofluorescence staining and quantitation showed a significant (*p* < 0.001) elevation of cPLA_2_α protein expression in the spinal cord (Fig. 1a) of 6-week-old hmSOD1 mice, before the loss of motor neuronal function that appeared around week 17 (Fig. 1c). cPLA_2_α upregulation was also detected in the mice brainstem from week 6 onward and showed similar results (not shown). Neuronal or glial cells were stained by specific markers in the spinal cord of hmSOD1 mice at the same time points (Fig. 1d). Immunofluorescence staining and quantitation of neurons in the spinal cord using anti-NeuN showed a significant (*p* < 0.001) reduction in the neuron cell area from week 19 that was further reduced at the end stage. The neurons were counted according to their size and we found a similar reduction of around 40 % at 19 weeks and around 60 % at end stage in each group, suggesting that the reduction in cell area is due to both the number and the size of the neurons. A significant (*p* < 0.001) appearance of activated microglia (M1 phenotype) could be detected in the spinal cord of 15-week-old hmSOD1 mice by Iba-1 or CD40 staining that was gradually increased with age. The activation of microglia at week 15 preceded neuronal damage (Fig. 1d) and the appearance of motor neuron dysfunction (Fig. 1c). Activated astrocytes determined by GFAP were significantly (*p* < 0.001) detected in the spinal cord of 19-week-old mSOD1 mice and were dramatically increased at the end stage. Double staining with Iba-1 and anti-CD169 for the detection of monocytes revealed a significant (*p* < 0.001) appearance of monocytes in the spinal cord only at the end stage (Fig. 1e). As shown by confocal microscopy in Fig. 2a and in the inset Fig. 2b, double staining analysis of cPLA_2_α and the different cell types suggests that the main cells expressing cPLA_2_α protein are the neurons. High levels of cPLA_2_α protein were detected in the large neurons present in the spinal cord of mSOD1 mice at 6 and 11 weeks and in the small and damaged neurons at 19 weeks and end stage. The slight reduction of cPLA_2_α protein expression at the end stage (Fig. 1a) is probably due to the reduced number and size of the neurons (Fig. 1d) which are the main cells expressing high levels of cPLA_2_α. Confocal microscopy of higher magnification of the double staining analysis of cPLA_2_α and Iba-1 or GFAP showed that elevated cPLA_2_α could be detected in activated microglia and astrocytes (Fig. 2b). Confocal microscopy of double staining of the activated form of cPLA_2_α, phospho-cPLA_2_α on serine 505 (pcPLA_2_α), and the markers of the different cell types (Fig. 2c) showed that pcPLA_2_α was detected in the nuclei of the cells.

**Fig. 3:**
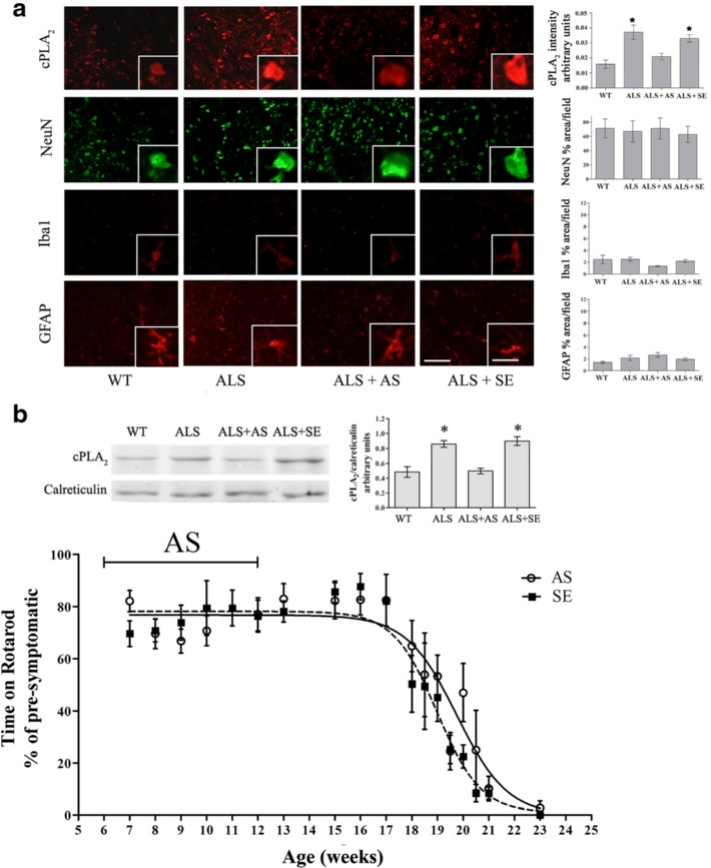
Reduction of cPLA_2_α upregulation at the early stage did not affect the development of the disease. Mice were brain infused with 10 μg/day AS (*n* = 12) or the corresponding sense (*n* = 6) or saline (*n* = 6) for 6 weeks starting at 6-week-old hmSOD1 mice. **a** Representative immunofluorescence staining of cPLA_2_α, neurons (NeuN), microglia (Iba-1), and astrocytes (GFAP) in the brain stem tissue section of WT mice, hmSOD1 mice (ALS), hmSOD1 mice brain infused with AS (ALS + AS) or sense (ALS + SE) at 12 weeks. Mice receiving saline or sense showed similar results and were combined under the sense-treated group. A double staining of cPLA_2_α and NeuN is presented. Scale bars = 50 μm, and in the insets = 20 μm. Shown are representative images of 12 mice in each group. The mean ± SEM of the fluorescence intensity (for cPLA_2_α) and percentage of the cell area (for cell markers) are presented in the bar graph. *p < 0.001—significance from WT mice and from AS-treated hmSOD1 mice. **b** A representative immunoblot analysis of cPLA_2_α and the corresponding calreticulin protein expression in the spinal cord lysates of the mice. cPLA_2_α protein expression was determined by dividing the intensity of each cPLA_2_α by the intensity of the corresponding calreticulin band after quantitation by densitometry and expressed in the bar graph as arbitrary units. The bar graph is the mean ± SE of 8 mice in each group. *p < 0.001—significance from WT mice and from AS-treated hmSOD1 mice. **c** Motor function by rotarod test of AS or sense brain infused-hmSOD1 mice (*n* = 12 in each group)

To determine whether cPLA_2_α has a role in the induction of the disease, its expression was blunted in the brain and spinal cord by means of specific oligonucleotide antisense against cPLA_2_α at 6-week-old hmSOD1 mice, when the elevation of cPLA_2_α was first detected. Ten micrograms per day of AS or the corresponding sense or saline was continuously pumped into the right lateral ventricle as done in our earlier study in a mouse model of amyloid beta brain infusion [19]. Using this methodology, it was shown [27] that significant oligonucleotide concentrations were achieved in the brain and brainstem and in all levels of the spinal cord. AS brain infusion to 6-week-old mice over a period of 6 weeks significantly prevented upregulation of cPLA_2_α in the brainstem as detected by immunofluorescence (Fig. 3a) and in the spinal cord as detected by Western blot analysis (Fig. 3b). As expected, at 12 weeks, there was no neuronal damage as well as no activation of microglia or astrocytes and the AS brain infusion had no effect (Fig. 3a). This treatment that did prevent the initial elevation of cPLA_2_α had no effect on the development of the disease assayed by motor function on rotarod (Fig. 3c). The initial value of 80 % of pre-symptomatic is due to the pump implantation surgery.

**Fig. 4:**
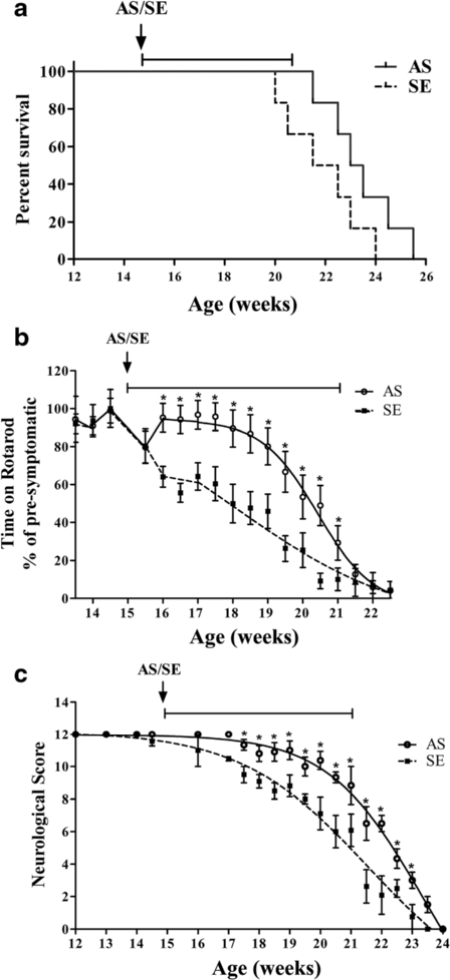
AS brain infusion to hmSOD1 mice shortly before the onset of motor neuron dysfunction delayed development of the disease. Fifteen-week-old hmSOD1 mice were brain infused with 10 μg/day AS or the corresponding sense during 6 weeks (*n* = 12 in each group). AS brain infusion prolonged survival (**a**), delayed loss of motor function analyzed by Rotarod (**b**), and delayed neurological score analyzed by a ladder (**c**). *p < 0.001—significance from sense-treated hmSOD1 mice

To determine the role of cPLA_2_α in the progression of the disease, 10 μg/day AS or sense was brain infused at week 15 (shortly before the onset of motor neuronal dysfunction) for 6 weeks (due to the pump’s limit). As shown in Fig. 4a, AS brain infusion prolonged the survival in mice by 12 days (<0.05). AS brain infusion significantly delayed the onset of motor neuron dysfunction by 3 weeks and there was a significant (*p* < 0.001) difference between the two groups until 21 weeks of age (Fig. 4b). Immediately after the pump implantation, there was a reduction in motor function, which was recovered only in the AS-treated hmSOD1 mice. The evaluation of the overall motor symptoms change in mice done by Ladder test (Fig. 4c) showed also a delay of 3 weeks in the loss of motor neuron function in the AS-treated hmSOD1 mice and a significant (*p* < 0.001) difference from the sense-treated hmSOD1 mice until 23 weeks of age.

**Fig. 5:**
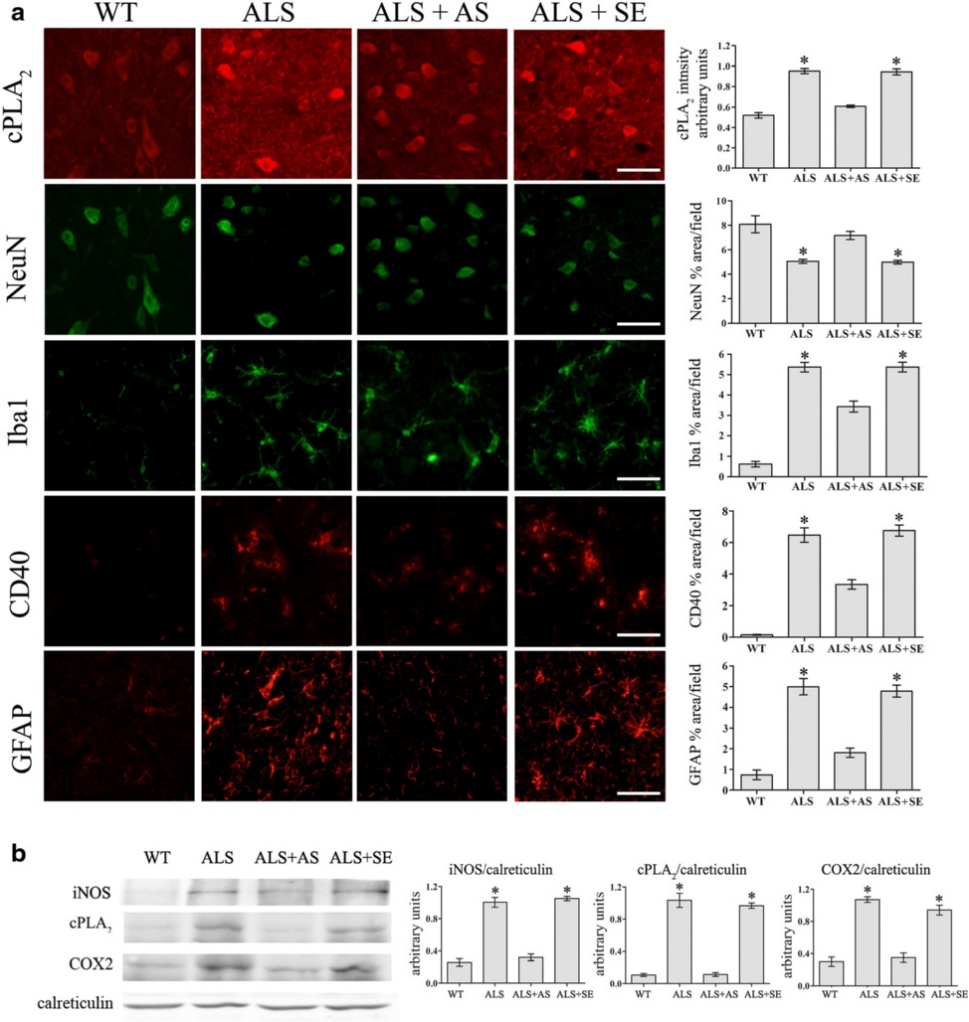
Reduction of cPLA_2_α upregulation in the spinal cord of hmSOD1 mice shortly before the onset of motor dysfunction reduced neuronal death and gliosis. Fifteen-week-old hmSOD1 mice were brain infused with 10 μg/day AS or the corresponding sense for 3–4 weeks and analyzed at week 18–19. **a** Representative immunofluorescence staining of cPLA_2_α, neurons (NeuN), microglia (Iba-1), and astrocytes (GFAP) in the spinal cord tissue section of WT mice, hmSOD1 mice (ALS), hmSOD1 mice brain infused with AS (ALS + AS) or sense (ALS + SE) at 18–19 weeks. Scale bars = 50 μm. Shown are representative images of 12 mice in each group. The mean ± SEM of the fluorescence intensity (for cPLA_2_α) and percentage of the cell area (for cell markers) are presented in the bar graph. *p < 0.001—significance from WT mice and from AS-treated hmSOD1 mice. **b** Representative immunoblot analysis of cPLA_2_α, COX-2, and iNOS and the corresponding calreticulin protein expression in the spinal cord lysates of the mice. Densitometry analysis for the three proteins was done as described for cPLA_2_α in Fig. 3b. The bar graph is the mean ± SE of 8 mice in each group. *p < 0.001—significance from WT mice and from AS-treated hmSOD1 mice

To determine the processes that are affected by cPLA_2_α during the development of the disease symptoms and to characterize the effect of prevention of cPLA_2_α upregulation on the different processes taking place at the first phase of development of motor neuron dysfunction, hmSOD1 mice were brain infused with AS or sense at week 15 for 3–4 weeks and the experiments were terminated for analysis at 18–19 weeks of age, at a time when the two mouse groups—AS and sense control—exhibited a significant difference in the disease symptoms. Non-treated hmSOD1 mice and WT mice at the same age were also studied. As shown in the immunofluorescence analysis and quantitation in Fig. 5a, cPLA_2_α protein expression was much lower in the spinal cord of 18–19-week-old AS-treated hmSOD1 mice in comparison with hmSOD1 mice or sense-treated hmSOD1 mice. AS treatment prevented the reduction in the number of the neurons (detected by NeuN), prevented astrocyte activation (detected by GFAP), and inhibited microglia activation (detected by Iba-1 and by CD40) compared with hmSOD1 mice or sense-treated hmSOD1 mice. In AS-treated hmSOD1 mice, reduction of cPLA_2_α upregulation led to diminution of the key inflammatory enzymes; iNOS and COX-2 as detected by Western blot analysis in the spinal cord lysates (Fig. 5b).

## Discussion

We show herein that cPLA_2_α is upregulated in the spinal cord of 6-week-old mice long before the appearance of the disease symptoms, neuronal death, or gliosis and remained elevated during the whole life span of the hmSOD1 mice. Prevention of cPLA_2_α upregulation at the pre-symptomatic period (between 6 and 12 weeks) did not affect the course of the disease. However, prevention of cPLA_2_α upregulation shortly before the onset of the disease symptoms significantly (*p* < 0.001) delayed the loss of motor neuronal function, suggesting that cPLA_2_α upregulation in the spinal cord plays a role in the disease pathology. In line with our results, the role of elevated spinal cord cPLA_2_α in neuronal damage was reported also in spinal cord injury [28] and in spinal inflammatory hyperalgesia [29].

In the present study we used, specific antisense brain infusion in order to determine the specific role of cPLA_2_α upregulation in the spinal cord in the pathogenesis of ALS. The advantage of the AS technique over knockout mice is its ability to inhibit the induction of an accelerated protein at the site of inflammation, and enables its expression at basal levels, whereas in knockout mouse models total elimination of a protein may result in dramatic changes of homeostasis and/or compensation by upregulation of other proteins (with similar biological features). The use of AS strategy is reinforced by two recent studies in cPLA_2_α knockout mice reporting that total elimination of cPLA_2_α resulted with abnormalities in architecture of synapses and cortical neurons [30] as well as in alteration of brain phospholipid composition [31]. AS brain infusion to 15-week-old hmSOD1 mice (for 6 weeks) shortly before the development of motor dysfunction resulted in reduction of cPLA_2_α elevated protein expression (as detected at 18–19 weeks in the spinal cord) and significantly delayed the development of the disease symptoms. This blunting of cPLA_2_α upregulation resulted in inhibition of activation of microglia and astrocytes as well as inhibition of motor neuronal death, as detected by immunofluorescence analysis in the spinal cord of 18–19-week-old hmSOD1 mice.

A generation of mice expressing a conditional deletion of the mutant SOD1 gene showed that each individual cell types in the spinal cord contributes to the development of ALS [8, 32]. Likewise, replacing the myeloid lineage of mutant SOD1 mice with wild-type microglia slowed disease progression [6]. Since cPLA_2_α upregulation was detected in each cell type in the spinal cord, it probably contributes by different mechanisms to motor neuron dysfunction and cell death: (i) elevated neuronal cPLA_2_α can act directly in inducing neuronal death (ii) while elevated glial cPLA_2_α can act indirectly by activating microglia and/or astrocytes. In our recent study in primary neuronal cultures, we reported that cPLA_2_α activation and upregulation induced apoptotic neuronal death [18]. Concomitant with our results, cDNA microarray analysis to monitor gene expression in the spinal cord of hmSOD1 mice during neurodegeneration revealed changes in apoptosis-related gene expression [4]. In addition, the role of cPLA_2_α in glutamate excitotoxicity and oxidative stress-induced cell death in primary spinal cord neuron cultures was recently reported [33]. Taken together the studies in neuronal cultures and the abundant levels of cPLA_2_α in motor neurons in the spinal cord, it is suggested that neuronal cPLA_2_α plays a direct role in motor neuron cell death. Similar to other neurodegenerative diseases and acute CNS insults, one of the most striking hallmarks of ALS shared by familial and sporadic patients, as well as by rodent models, is neuro-inflammation, characterized by extensive microglial activation and astrogliosis that contribute to disease progression, rather than to its resolution [2, 34–36]. Reduction of cPLA_2_α upregulation in the spinal cord of the hmSOD1 mice prevented astrocyte activation detected by GFAP immunostaining that is in line with *in vitro* studies [37] reporting that activation of cPLA_2_α led to an increase in oxidative stress in astrocytes. We show here a massive activation of microglia in the spinal cord (detected by immunostaining of Iba-1 and CD40) that preceded the changes in the motor neurons, in accordance with other studies [6, 38, 39]. This microglia activation was shown to be cPLA_2_α dependent coincided with ours and others’ studies in cell cultures demonstrating the specific role of microglial cPLA_2_α in the activation and transformation of microglia to M1 phenotype. We have previously reported that cPLA_2_α activity regulated the production of superoxides by NOX-2 NADPH oxidase and the induction of COX-2 and iNOS *via* nuclear factor kB (NF-kB) in microglia cultures [17]. Interestingly, microglial NF-kB specifically has been shown to play a major role in the development of the ALS in hmSOD1 mice [40]. We show here that reduction of cPLA_2_α in the spinal cord also decreased iNOS and COX-2 upregulation that produce two major proinflammatory mediators; nitric oxide and PGE_2_, respectively. As shown in the present study for cPLA_2_α, it was also reported that in both early symptomatic and end-stage transgenic hmSOD1 mice, neurons and to a lesser extent glial cells in the spinal cord exhibit robust COX-2 [41] and iNOS immunoreactivity [42]. Likewise, similar to cPLA_2_α, COX-2 was dramatically increased in postmortem spinal cord samples from sporadic ALS patients [ 41]. Nitric oxide and superoxides both under cPLA_2_α regulation [17] can form the toxic reagent peroxynitrite [43, 44]. In this context, a recent study reported that inhibition of cPLA_2_α activity ameliorated experimental autoimmune encephalomyelitis *via* blocking peroxynitrite formation in mouse spinal cord white matter [45]. These results suggest that glial cPLA_2_α is involved by different inflammatory processes in the development of ALS. In accordance with this suggestion, injections of PLA_2_ into the spinal cord of mice caused inflammation and oxidative stress [46].

The cause of cPLA_2_α elevation in the brain and spinal cord as early as 6-week-old mice is not yet clear. SOD1 insoluble protein complexes (IPCs) were detected in motor neurons of 30-day-old mSOD1 mice [47], before the manifestation of ALS pathology, and several months before the appearance of inclusion bodies. It may be possible that the formations of IPCs in the neurons triggered the elevation of cPLA_2_α. The role of this early elevation of cPLA_2_α is also not known and prevention of this elevation for 6 weeks had no effect on the course of the disease.

The role of monocyte recruitment to the spinal cord in ALS is under debate. On one hand, it was reported that inhibition of monocyte recruitment to the spinal cord delayed motor dysfunction and increased the survival of ALS mice [48]. In contrast, another study [49] reported that monocytes could not be detected in the spinal cord of SOD1G93A mice and that microglia are not derived from infiltrating monocytes. Likewise, a recent study [50] reported that in the mutant SOD1G93A mouse model of ALS, the choroid plexus of the brain did not support leukocyte trafficking during disease progression, due to a local reduction in IFN-γ levels. The results of the present study, in agreement with the two later reports, show that the recruitment of monocytes detected by co-immunostaining with CD169 and Iba-1 was evident only at the end stage.

## Conclusions

We show here that cPLA_2_α is elevated at 6 weeks, long before the appearance of the disease symptoms. cPLA_2_α elevation at the onset of symptoms plays a role in the induction of motor dysfunction. Reduction of cPLA_2_α upregulation shortly before the loss of motor neurons function significantly delayed the appearance of the disease symptoms. Elevated neuronal and glial cPLA_2_α may contribute by different aspects involved in the pathogenesis of ALS. Thus, cPLA_2_α may offer an efficient target for treatment of ALS and potentially other multifactorial neurodegenerative diseases.

## Abbreviations

ALS, amyotrophic lateral sclerosis; AS, antisense oligonucleotide against cPLA_2_α; C9ORF72, chromosome 9 open reading frame 72; COX-2, cyclooxygenase-2; cPLA_2_α, cytosolic phospholipase A_2_ alpha; hmSOD1, human mutant SOD1G93A; iNOS, inducible nitric oxide synthase; NF-kB, nuclear factor kB; SOD1, superoxide dismutase; TDP-43, TAR DNA binding protein
